# First isolation of West Nile virus from a dromedary camel

**DOI:** 10.1038/emi.2016.53

**Published:** 2016-06-08

**Authors:** Sunitha Joseph, Ulrich Wernery, Jade LL Teng, Renate Wernery, Yi Huang, Nissy AG Patteril, Kwok-Hung Chan, Shyna K Elizabeth, Rachel YY Fan, Susanna KP Lau, Jörg Kinne, Patrick CY Woo

**Affiliations:** 1 Central Veterinary Research Laboratory, Dubai, the United Arab Emirates; 2 Department of Microbiology, The University of Hong Kong, Hong Kong, China; 3 State Key Laboratory of Emerging Infectious Diseases, The University of Hong Kong, Hong Kong, China; 4 Research Centre of Infection and Immunology, The University of Hong Kong, Hong Kong, China; 5 Carol Yu Centre for Infection, The University of Hong Kong, Hong Kong, China; 6 Collaborative Innovation Center for Diagnosis and Treatment of Infectious Diseases, Zhejiang University, Hangzhou 310000, Zhejiang Province, China

**Keywords:** west Nile virus, dromedary camel, United Arab Emirates

## Abstract

Although antibodies against West Nile virus (WNV) have been detected in the sera of dromedaries in the Middle East, North Africa and Spain, no WNV has been isolated or amplified from dromedary or Bactrian camels. In this study, WNV was isolated from Vero cells inoculated with both nasal swab and pooled trachea/lung samples from a dromedary calf in Dubai. Complete-genome sequencing and phylogenetic analysis using the near-whole-genome polyprotein revealed that the virus belonged to lineage 1a. There was no clustering of the present WNV with other WNVs isolated in other parts of the Middle East. Within lineage 1a, the dromedary WNV occupied a unique position, although it was most closely related to other WNVs of cluster 2. Comparative analysis revealed that the putative E protein encoded by the genome possessed the original WNV E protein glycosylation motif NYS at E154–156, which contained the *N*-linked glycosylation site at N-154 associated with increased WNV pathogenicity and neuroinvasiveness. In the putative NS1 protein, the A70S substitution observed in other cluster 2 WNVs and P250, which has been implicated in neuroinvasiveness, were present. In addition, the *foo* motif in the putative NS2A protein, which has been implicated in neuroinvasiveness, was detected. Notably, the amino-acid residues at 14 positions in the present dromedary WNV genome differed from those in most of the closely related WNV strains in cluster 2 of lineage 1a, with the majority of these differences observed in the putative E and NS5 proteins. The present study is the first to demonstrate the isolation of WNV from dromedaries. This finding expands the possible reservoirs of WNV and sources of WNV infection.

## INTRODUCTION

West Nile virus (WNV) is a positive-sense single-stranded ribonucleic acid (RNA) virus in the Flaviviridae family. WNV is the leading cause of mosquito-borne encephalitis in humans in many parts of the world. In addition to humans, many animals are also susceptible to WNV infections. Large outbreaks have occurred globally in both humans and other animals, such as horses and pigs. Birds are the natural reservoirs of WNV. Bird-to-bird, bird-to-mammal and bird-to-human transmissions are achieved by mosquito bites, with humans and other mammals serving as dead-end hosts because of low viral loads.

Camels are one of the most unique mammals on earth and have shown perfect adaptation to desert life. There are two surviving old-world camel species: *Camelus dromedarius* (dromedary or one-humped camel), which inhabits the Middle East and North and Northeast Africa, and *Camelus bactrianus* (Bactrian or two-humped camel), which inhabits Central Asia. Among the 20 million camels on earth, 90% are dromedaries. Although WNV is known to infect some of the new world camels, such as llamas and alpacas,^1^ only antibodies against WNV have been detected in the sera of old-world camels, such as the dromedaries in the Middle East, North Africa and Spain, with a seroprevalence of 3%–38%.^2, 3, 4, 5^ To date, no WNV has been isolated or amplified from either dromedary or Bactrian camels. Recently, the emergence of Middle East respiratory syndrome (MERS) and the isolation of the MERS coronavirus (MERS-CoV) from dromedaries boosted interest in the search for novel viruses in dromedaries.^6, 7, 8, 9, 10, 11, 12, 13^ In this article, we report the first isolation of WNV from a dromedary calf in the United Arab Emirates during the process of MERS-CoV screening and the results of the comparative genome and phylogenetic analysis.

## MATERIALS AND METHODS

### Sample collection and viral culture

Clinical samples were obtained during a necropsy of a dromedary calf at the Central Veterinary Research Laboratory in Dubai, the United Arab Emirates, using standard procedures. The Central Veterinary Research Laboratory in Dubai is the center for performing necropsies of dromedaries from sheikhs in the UAE with the aim of finding the cause of death and preventing the spread of infectious diseases to other camels or herds. Nasal swabs and pooled ground trachea/lung samples were inoculated onto Vero cells for MERS-CoV screening.^14^ The pooled clinical samples were diluted 10-fold with viral transport medium and filtered. Two hundred microliters of the filtrate was inoculated into 200 μL of minimum essential medium (Gibco, Grand Island, NY, USA). Four hundred microliters of the mixture was added to 24-well tissue culture plates with Vero cells by adsorption inoculation. After 1 h of adsorption, the excess inoculum was discarded, the wells were washed twice with phosphate-buffered saline, and the medium was replaced with 1 mL of minimum essential medium (Gibco). Cultures were incubated at 37 °C with 5% CO_2_ and inspected for cytopathic effects daily using inverted microscopy.

### Electron microscopy

Negative-contrast electron microscopy was performed as previously described.^15^ Tissue culture cell extracts infected with dromedary WNV were centrifuged at 19 000*g* at 4 °C. Then, the pellet was resuspended in phosphate-buffered saline and stained with 2% phosphotungstic acid. Samples were examined with a Philips EM208s electron microscope (Philips Scientifics, Eindhoven, The Netherlands).

### Sample preparation for Illumina sequencing

RNA was extracted from the isolated virus using the QIAamp Viral RNA Mini Kit (Qiagen, Hilden, Germany). Reverse transcription and polymerase chain reaction (PCR) were performed using the SuperScript III reverse transcriptase (Invitrogen, Carlsbad, CA, USA) and a random primer containing a 20-base arbitrary sequence at the 5′ end followed by a randomized octamer (8*N*) at the 3′ end. A single round of priming and extension was performed using the Klenow fragment polymerase (New England Biolabs, Ipswich, UK). PCR amplification with a primer consisting of only the 20-base arbitrary sequence of the random primer was performed with 20 cycles of 94 °C for 15 s, 60 °C for 30 s and 68 °C for 1 min and a final extension at 68 °C for 7 min in an automated thermal cycler (Applied Biosystems, Carlsbad, CA, USA). Standard precautions were taken to avoid PCR contamination, and no amplified PCR product was observed in the negative control. The PCR product was purified using the MinElute PCR Purification Kit (Qiagen) following the manufacturer's protocol. The purified DNA was eluted in 15 μL of EB buffer and used as the template for library construction.^16^

### Library construction for Illumina sequencing

The metagenomics library was generated using a DNA library prepared with the Nextera XT DNA sample preparation Kit (Illumina, San Diego, CA, USA) according to the manufacturer's protocol. Briefly, 1 ng of input DNA was tagmented by the Nextera XT transposome at 55 °C for 5 min. This transposome simultaneously fragmented the input DNA and added an adapter sequence to the ends, thereby allowing amplification by PCR in subsequent steps. The sequencing library with the tagmented DNA was amplified in 12 cycles of 95 °C for 10 s, 55 °C for 30 s and 72 °C for 30 s and a final extension at 72 °C for 5 min in an automated thermal cycler (Applied Biosystems). The amplified DNA library was purified using 1.8 × AMPure XP beads (Beckman Coulter, Indianapolis, IN, USA) to remove very short library fragments from the population. The amplified DNA library was analyzed using the 2100 Bioanalyzer instrument (Agilent Technologies, Santa Clara, CA, USA). The purified sequencing library was quantified using the KAPA library quantification kit (Kapa Biosystems, Wilmington, DE, USA) and subsequently sequenced on the Illumina HiSeq 2500 with 151 bp paired-end reads (Rapid Run Mode). Image analysis and base calling were performed with SCS2.8/RTA1.8 (Illumina). FASTQ file generation and the removal of failed reads were performed using CASAVA ver.1.8.2 (Illumina).^16^

### Genome assembly and complete-genome sequencing

Illumina sequence reads were quality trimmed by Prinseq-lite, and *de novo* genome assembly was performed with MIRA 4.9. The 5′ and 3′ ends of the viral genome were confirmed by rapid amplification of cDNA ends (RACE) using the 5′/3′ RACE kit (Roche, Penzberg,Germany).

### Comparative genome analysis and phylogenetic analysis

The nucleotide sequence of the genome and the deduced amino-acid sequences of the open reading frames were compared with those of other WNV strains with complete genomes in the ViPR sequence database (http://www.viprbrc.org/). *N*-linked glycosylation sites were predicted using the NetNGlyc 1.0 software (http://www.cbs.dtu.dk/services/NetNGlyc/). Complete polyprotein nucleotide sequences were aligned with MUSCLE. A maximum-likelihood phylogenetic tree with 1000 bootstraps was constructed using the GTR substitution model; gamma distribution among sites was conducted in MEGA5.

### Nucleotide sequence accession numbers

The nucleotide sequence of the dromedary WNV genome isolated in this study has been lodged in the GenBank sequence database under accession NO KU588135.

## RESULTS

### Necropsy, virus culture and electron microscopy

**Figure 1:**
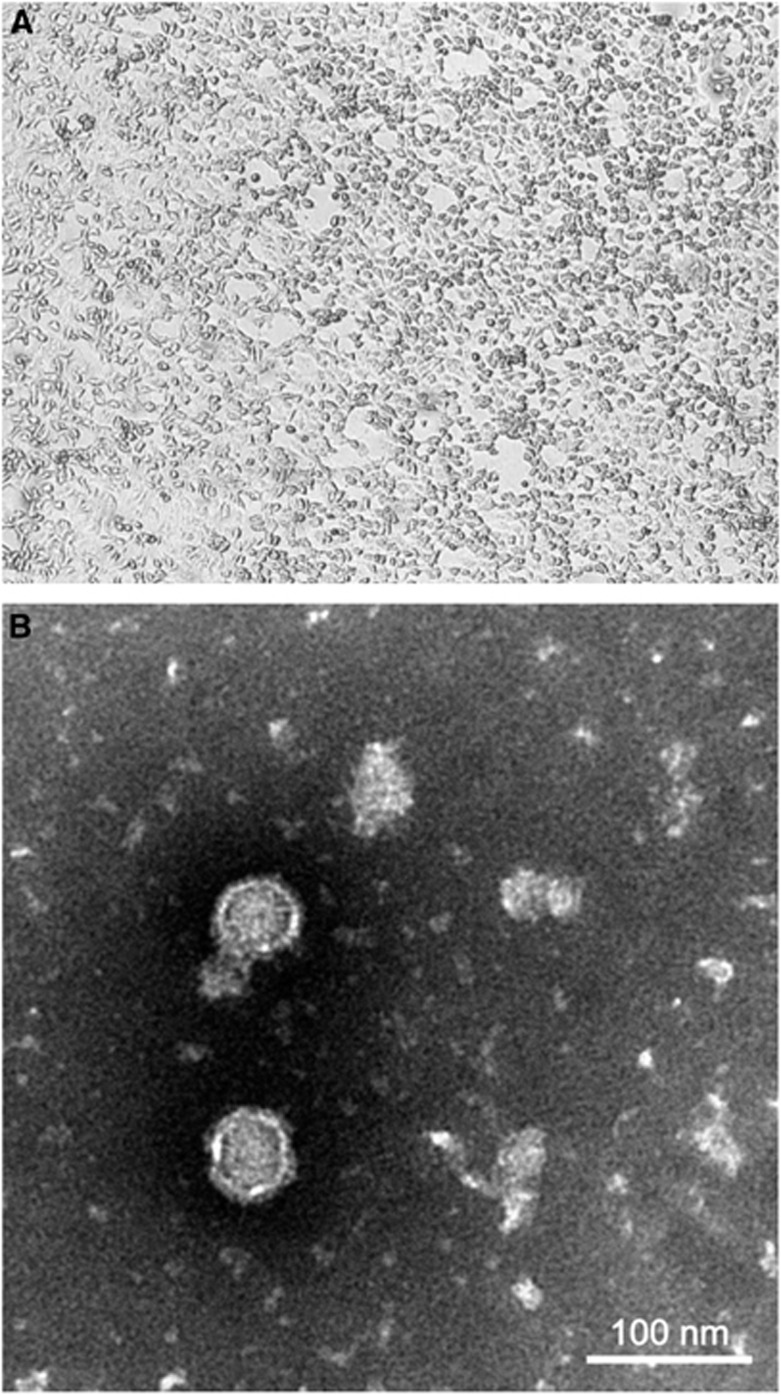
(**A**) Cytopathic effects of WNV from a dromedary on Vero cells, showing cell rounding, degeneration and detachment, magnification: × 40. (**B**) Negative contrast electron microscopic examination of an infected Vero cell culture supernatant showing enveloped icosahedral viral particles, bar=100 nm.

Necropsy of the female 1-month-old 50 kg fresh dromedary calf revealed pale muscles and myocard, massive lung congestion and colitis. The causes of death were white muscle disease, colisepticemia and *Candida* enteritis. At the first passage, the Vero cells inoculated with both the nasal swab and pooled trachea/lung samples showed identical cytopathic effects on day 4, with cell rounding, progressive degeneration and detachment (Figure 1A). Electron microscopy showed enveloped icosahedral virions 45–50 nm in diameter with a 25- to 35-nm electron dense core (Figure 1B). Both the cytopathic effects and viral morphology were inconsistent with those of MERS-CoV.

### Complete-genome sequencing and genome structure

The complete genome of the isolated virus was sequenced and assembled. The size, G+C content and genome structure were similar to other WNVs. The single polyprotein is putatively cleaved by proteases into three structural proteins (capsid (C), premembrane/membrane (prM/M) and envelope (E)) and seven nonstructural proteins (NS1, NS2A, NS2B, NS3, NS4A, NS4B and NS5).

### Phylogenetic analysis

**Figure 2:**
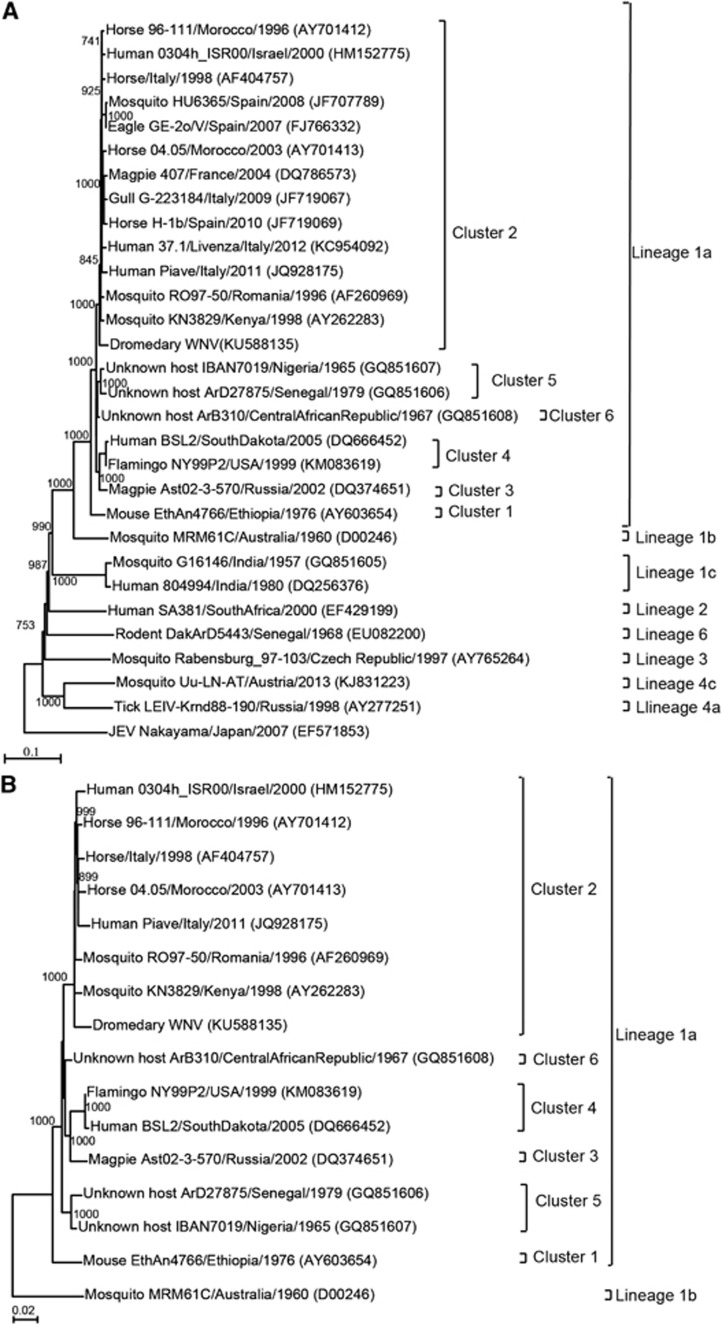
Phylogenetic tree showing the relationship of dromedary WNV to other WNV strains based on a complete polyprotein sequence by the maximum-likelihood method in MEGA5. (**A**) WNV strains of all lineages and clusters are indicated except for those of lineages 4b, 5 and 7 because no complete polyprotein sequence is available from these lineages. A total of 10 345 nucleotide positions were included in the analysis. Japanese encephalitis virus strain Nakayama (EF571853) was used as the outgroup. (**B**) WNV strains of cluster 2 that are most related to the dromedary WNV and strains of the other clusters within lineage 1a are indicated. A total of 10 302 nucleotide positions were included in the analysis. Kunjin strain MRM61C (D00246) of lineage 1b was used as the outgroup. The scale bar indicates the number of nucleotide substitutions per site. All names and accession numbers are given as cited in the ViPR sequence and GenBank databases. Bootstrap values are shown only for nodes that were well supported by the maximum-likelihood method (⩾700 bootstrap support).

Phylogenetic analysis using the near-whole-genome polyprotein revealed that the virus belonged to lineage 1a (Figure 2A).^17^ The present dromedary WNV occupied a unique position within lineage 1a, although it was most closely related to other WNVs of cluster 2 (Figure 2B).

### Comparative genome analysis

**Figure 3:**
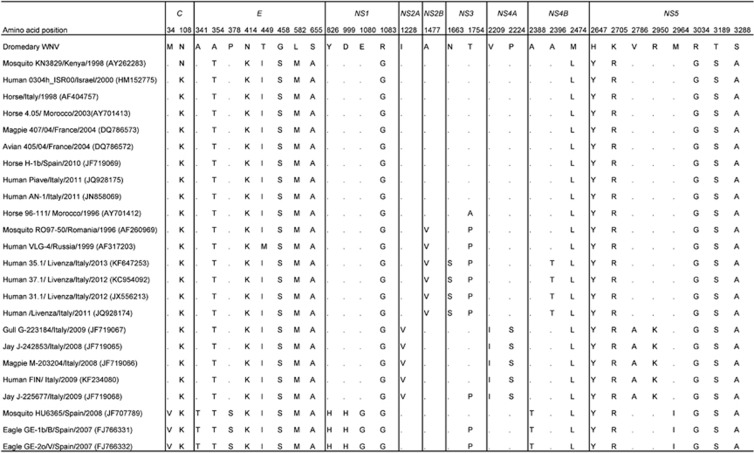
Summary of amino-acid changes in the various putative proteins encoded by the dromedary WNV genome relative to those of its most closely related WNV strains in cluster 2 of lineage 1a. Only changes that occurred in ⩾3 strains are shown. Dots indicate no difference from the dromedary WNV.

Comparative analysis based on the complete polyprotein sequence showed the highest (99.51%–99.56%) overall amino-acid identities to WNV strains isolated from a mosquito in Kenya (Genbank accession number AY262283), horses in Italy and Morocco (Genbank accession numbers AF404757, AY701413 and AY701412) and a human in Italy (Genbank accession number JQ928175). Detailed annotation revealed that the putative E protein encoded by the genome possessed the original WNV E protein glycosylation motif NYS at E154–156, which contained the *N*-linked glycosylation site at N-154 associated with increased WNV pathogenicity and neuroinvasiveness.^18^ In the putative NS1 protein, the A70S substitution observed in other cluster 2 WNVs and P250, which was implicated in neuroinvasiveness, were also present.^19^ In addition, the *foo* (flavivirus overlapping ORF) motif in the putative NS2A protein that was implicated in neuroinvasiveness was detected.^20^ Notably, 14 amino-acid residues in the present dromedary WNV genome differed from those in most of the closely related WNV strains in cluster 2 of lineage 1a (Figure 3), with the majority of these differences observed in the putative E and NS5 proteins.

## DISCUSSION

We report the first isolation of WNV in a dromedary. The first evidence for the presence of hemagglutination inhibiting and complement-fixing antibodies in dromedaries from Nigeria against WNV was published in 1990.^21, 22^ Recently, a study has shown the presence of neutralizing antibodies against WNV from dromedaries in North Africa.^3^ In the United Arab Emirates, antibodies against WNV have been reported in 38% of 1119 dromedary sera^5^ and 19.2% of 750 tested equine sera,^23^ supporting the conclusion that WNV is present in the country. In the present study, we isolated a WNV from two independent respiratory samples from a dromedary calf using Vero cells, which is one of the most widely used cell lines for the recovery of WNV. This finding was consistent with previous studies that showed that WNV could also be detected in respiratory specimens from birds, humans and other mammals.^24, 25^ Notably, the glycosylation motif NYS in the E protein, the A70S substitution and P250 in the NS1 protein and the *foo* motif in the NS2A protein, which are important for pathogenicity and neuroinvasiveness, were also detected in the present WNV isolate.^18, 19, 20^ However, because there were no clinical signs in the dromedary calf, the pathogenic role of WNV in dromedaries remains to be determined. Viral cultures using other samples from the dromedary were not performed because the samples were initially intended for MERS-CoV isolation.

The present dromedary WNV belongs to WNV lineage 1a. The whole-genome phylogenetic trees showed that the present dromedary WNV was most closely related to other WNVs in cluster 2 of lineage 1a with high bootstrap support (Figure 2). However, there was no clustering of the present WNV with WNVs isolated in other parts of the Middle East, such as Israel, Egypt and Cyprus (data not shown). Indeed, two WNVs from Israel (Genbank accession number AY688948) and Cyprus (Genbank accession number GQ903680) belong to lineage 2, another isolate from Israel (Genbank accession number HM152773) belongs to cluster 4 of lineage 1a, and two other isolates from Israel (Genbank accession number HM051416) and Egypt (Genbank accession number EU081844) belong to cluster 1 of lineage 1a. Moreover, 14 amino-acid positions located in various regions of the genome encoding five different proteins showed marked differences with the corresponding amino acids in the genomes of the isolate's close relatives in cluster 2. Interestingly, most of the amino-acid differences between the present dromedary WNV and the closely related WNVs from Africa and Europe are located in the E and NS5 genes. The E protein is generally the least conserved protein because it is exposed to external selection pressure, whereas the NS5 protein is highly conserved because it contains the viral methyltransferase and RNA-dependent RNA polymerase. Complete-genome sequencing of more WNV strains and comparative genomic and phylogenetic studies are needed to ascertain whether dromedary WNVs form a unique cluster in lineage 1a.

The MERS epidemic and the discovery of dromedaries as the reservoir of MERS-CoV have boosted interest in the search for novel viruses in dromedaries. Prior to 2013, viruses from at least eight families were found to infect camels. In the last two years, we have reported the discovery of a novel dromedary camel CoV (UAE-HKU23), a novel genotype of hepatitis E virus, a novel genus of enterovirus, a novel astrovirus, and novel picobirnaviruses and circoviruses in dromedaries.^16, 26, 27, 28, 29^ These discoveries have remarkably widened the spectrum of viruses known to infect dromedaries. Notably, after the description of the novel genotype of hepatitis E virus in dromedaries, another group found this dromedary camel hepatitis E virus as a cause of chronic hepatitis E infection in a liver transplant recipient with a habit of camel milk and meat consumption, highlighting the importance of camels as a source of infectious diseases.^30^ The present study is the first demonstration of the isolation of WNV in dromedaries and the first WNV complete-genome sequenced from a dromedary among the relatively few full-length WNV genome sequences from the Middle East. Further studies will help elucidate whether dromedaries can serve as another possible source of WNV infection and determine the tissue tropism of WNV in dromedaries. Other studies on the epidemiology, disease spectrum and ecology of WNV in this group of unique animals are also warranted.
